# NK Cell Isolation from Liver Biopsies: Phenotypic and Functional Analysis of Low Cell Numbers by Flow Cytometry

**DOI:** 10.3389/fimmu.2013.00061

**Published:** 2013-03-11

**Authors:** Ning Li, Gisella L. Puga Yung, Amandine Pradier, Christian Toso, Emiliano Giostra, Isabelle Morard, Laurent Spahr, Jörg D. Seebach

**Affiliations:** ^1^Division of Clinical Immunology and Allergology, Department of Medical Specialties, University Hospital and Medical FacultyGeneva, Switzerland; ^2^Division of Gastroenterology and Hepatology, Department of Medical Specialties, University Hospital and Medical FacultyGeneva, Switzerland; ^3^Division of Transplant and Visceral Surgery, Department of Surgery, University Hospital and Medical FacultyGeneva, Switzerland

**Keywords:** human NK cells, liver biopsy, intrahepatic lymphocytes, collagenase, flow cytometry, IFN-γ, CD107a

## Abstract

Natural killer (NK) cells are considered to play a critical role in liver disease. However, the available numbers of intrahepatic lymphocytes (IHL) derived from liver biopsies (LB) for *ex vivo* analysis of intrahepatic NK cells is very limited; and the isolation method may hamper not only yields and viability, but also phenotype and function of IHL. The aim of the present study was therefore to (1) refine and evaluate the cell yields and viability of a modified isolation protocol from standard size needle LB; and (2) to test the effects of mechanical dissociation and enzymatic tissue digestion, as well as the analysis of very low cell numbers, on the phenotype and function of intrahepatic NK cells. Peripheral blood mononuclear cells (PBMC) and IHL, freshly isolated from the peripheral blood, LB (*n* = 11) or partial liver resections (*n* = 5), were used for phenotypic analysis by flow cytometry. NK cell function, i.e., degranulation and cytokine production, was determined by staining of CD107a and intracellular IFN-γ following *in vitro* stimulation. The mean weight of the LB specimens was 9.1 mg, and a mean number of 7,364 IHL/mg were obtained with a viability of >90%. Exposure of IHL and PBMC to 0.5 mg/ml collagenase IV and 0.02 mg/ml DNase I for 30 min did affect neither the viability, NK cell function, nor the percentages of CD56^+^, NKp46^+^, and CD16^+^ NK cells, whereas the level of CD56 surface expression was reduced. The phenotype of LB-derived NK cells was reliably characterized by acquiring as few as 2,500 IHL per tube for flow cytometry. The functional assay of intrahepatic NK cells was miniaturized by culturing as few as 25,000 IHL in 25 μl (10^6^/ml) using 96-well V-bottom plates with IL-2 and IL-12 overnight, followed by a 4 h stimulation with K562 cells at a NK:K562 ratio of 1:1. In summary, we report reliable phenotypic and functional analyses of small numbers of intrahepatic NK cells isolated from LB specimens providing us with a tool to better address the emerging role of human NK cell immunobiology in liver diseases.

## Introduction

The liver is a unique immunological site, constantly exposed to gut-derived bacterial products, environmental toxins, and food antigens (Crispe, 2009). The presence of a strong innate immune system provides rapid and efficient resistance against potential pathogens and toxic compounds, while tolerance mechanisms prevent harmful responses against food antigens and auto-immunity (Doherty and O’Farrelly, 2000; Gao et al., 2008). Beside the cells implicated in adaptive immunity, such as T lymphocytes and dendritic cells, several innate immune cells, such as natural killer (NK), NKT, γδ T cells, and Kupffer cells, are present in the liver (Gao et al., 2008; Yamagiwa et al., 2009).

Intrahepatic NK cells are preferentially located in the distinctive, thin-walled sinusoids, whereas Kupffer cells and resident lymphocytes are mostly found in the parenchyma (Doherty and O’Farrelly, 2000; Crispe, 2009). In humans, the repertoire of intrahepatic lymphocytes (IHL) differs from peripheral blood lymphocytes with NK cells comprising approximately 30–50% (of IHL) (Norris et al., 1998; Doherty and O’Farrelly, 2000; Tian et al., 2012), a much higher percentage than in the peripheral blood (5–15% of peripheral blood lymphocytes) (Lanier et al., 1986). Emerging data from observational human and experimental animal studies have revealed that the liver microenvironment modifies the activating/inhibitory NK cell receptor expression and the functional responsiveness of NK cells recruited to the liver. These modifications depend on both the secretion of cytokines and cell-to-cell interactions, such as the crosstalk between NK cells and liver-resident dendritic (Jinushi et al., 2007) and stellate cells (Hintermann et al., 2010). Thus, NK cells play a central role not only in innate immunity, but also in shaping adaptive immune response in the liver (Krueger et al., 2011). Taken together, intrahepatic NK cells seem to exhibit unique phenotypic and functional properties in health and disease (Norris et al., 1998; Ishiyama et al., 2006; Bonorino et al., 2009; Mondelli et al., 2010; Zhang et al., 2011) and have therefore become an object of great scientific interest, in particular with respect to their role in controlling viral hepatitis, liver fibrosis, and liver tumorigenesis but also in contributing to the pathogenesis of liver injury and persistent inflammation (Nair et al., 2010; Shi et al., 2011; Tian et al., 2012).

The great interest in intrahepatic NK cells is reflected by the multitude of recent reports on their phenotype (Nattermann et al., 2005, 2006; Dunn et al., 2007; Yamagiwa et al., 2008; Ahlenstiel et al., 2010; Mondelli et al., 2010). So far, the source for these studies has mainly been liver resection specimens thus limiting the spectrum of patients accessible to analysis to those undergoing surgery. On the other hand, liver biopsies (LB) are the diagnostic “gold standard” for many liver pathologies, visualizing the necro-inflammatory and architectural status of the liver by immunohistology, with the advantage of precise *in situ* localization of IHL (Bravo et al., 2001; Cholongitas et al., 2006; Lefkowitch, 2007; Nair et al., 2010). Nevertheless, while much more practical and less invasive than liver resections, percutaneous or transjugular needle LB have a number of limitations for research purposes. First, some antibodies used for cell staining are only applicable by flow cytometry, but not by immunohistochemistry; second, it is demanding to stain for multiple immunohistological markers necessary for the identification of specific IHL cell subsets. Third, the small size of LB limits the number of IHL that can be isolated, and the mechanical and enzymatic isolation methods may hamper their viability, the cell yields and cell-surface expression of certain lymphocyte markers (Mulder et al., 1994; Ishiyama et al., 2006; Mondelli et al., 2010). Consequently, using LB specimens for detailed phenotypical analyses of IHL, and in particular for functional studies requiring *in vitro* culturing has been a challenging endeavor. For all these technical reasons the vast majority of functional studies of NK cell in liver diseases have focused on circulating peripheral blood NK cells rather than on intrahepatic NK cells. However, the results obtained with circulating NK cells do not necessarily predict the cytotoxicity or cytokine production of intrahepatic NK cells.

The aim of the present study was (1) to refine and evaluate a modified protocol to isolate IHL from standard size needle LB samples with respect to cell yields and viability, and (2) to test the effects of the isolation protocol and the analysis of very low cell numbers on phenotype and function of intrahepatic NK cells. To this end, intrahepatic NK cells isolated from LB and partial liver resection specimens were analyzed in comparison to peripheral blood NK cells. The ultimate goal is to use this flow cytometry-based method as a tool to perform detailed immunological analyses of the role of intrahepatic NK cells and other lymphocyte subsets in liver disease.

## Materials and Methods

### Reagents, media, and antibodies

The following reagents were used for cell isolation in this study: Ficoll-Paque (GE Healthcare), collagenase type IV (Catalog number C5138, 700.5 U/mg solid collagen, Sigma-Aldrich), DNase I grade II (Catalog number 10104159001, Roche), ACK lysing buffer (Lonza), bovine serum albumin (BSA), and sodium azide (both from Sigma-Aldrich). Buffer 1 was composed of Hank’s balanced salt solution (HBSS) supplemented with 0.5 mg/ml collagenase IV; 0.02 mg/ml DNase I; 2% fetal calf serum (FCS, from Sigma-Aldrich); 25 mM HEPES; and 0.6% BSA, whereas buffer 2 was composed of HBSS supplemented with 2% fetal calf serum (FCS, from Sigma-Aldrich), 25 mM HEPES, 0.6% BSA, and 0.01 mg/ml DNase.

Cell culture media AIM-V, RPMI 1640, HBSS, phosphate buffered saline (PBS), HEPES, penicillin/streptomycin, MEM non-essential amino acids, essential amino acids, and sodium pyruvate were from Gibco-BRL. l-Glutamine was from Biochrom AG. Heat-inactivated pooled human AB serum was obtained from the local blood transfusion center (Centre de transfusion sanguine, HUG). Recombinant interleukin-2 (IL-2) was from Novartis, and IL-12 from R&D Systems. Complete AIM-V medium refers to AIM-V supplemented with 10% heat-inactivated pooled human AB serum; 2 mM l-glutamine; 20 mM HEPES; 1 mM Na-pyruvate; 1% MEM non-essential amino acids; 2% essential amino acids; 1% penicillin–streptomycin; 50 U/ml of IL-2; and 0.5 ng/ml of IL-12.

Anti-CD3 PE-Cy7 (clone SK7), anti-CD107a PE (clone H4A3), and mouse-isotype IgG1 matched controls mAb (clone MOPC21) were obtained from BD Biosciences. Anti-CD3 FITC (clone UCHT1), anti-IFN-γ FITC (clone 4S.B3), anti-CD16 PE (clone 3G8), and anti-NKp46 PE (clone 9E2) were purchased from BioLegend. Anti-CD56 APC (clone AF12-7H3, 8) was from Miltenyi Biotec. BD Cytofix/Cytoperm and GolgiStop solutions were also from BD Bioscience. Staining buffer consisted of PBS supplemented with 0.1% BSA and 0.02% sodium azide. All antibodies were titrated appropriately in order to assure saturating conditions for 0.1 × 10^6^ cells or less using 50 μl incubation volumes. Titrations started at the manufacturers’ suggestions. Final dilutions varied from one antibody to another. The same amount of antibody was used for 0.1 × 10^6^ to 2,500 cells.

### Samples

All liver tissue donors were treated at the University Hospital of Geneva. The study protocol was approved by the institutional review board, and written informed consent was obtained from each subject. A total of 11 specimens were obtained from patients suffering from chronic liver diseases at the time of percutaneous or transjugular needle biopsy performed for diagnostic reasons. The age of the patients ranged from 34 to 63 years (median 51 years), and the male:female ratio was 5:6 (Table 1). Each sample was sent for routine histological assessment and only the remaining tissue was available for the isolation of IHL. In addition, five partial liver resection specimens were obtained from patients during surgery for hepatocellular carcinoma (*n* = 3), or echinococcosis (*n* = 2). All tumor nodules, necrotic, and infected tissue fractions were removed in order to obtain macroscopically unaffected liver samples. The specimens were collected in RPMI 1640 medium supplemented with 2 mM l-glutamine, 25 mM HEPES, 10% FCS, penicillin (50 U/ml), and streptomycin (50 μg/ml), and immediately transported to the laboratory for cell isolation. Peripheral blood mononuclear cells (PBMC) were isolated from the blood of healthy volunteers by standard Ficoll-Paque density gradient centrifugation separation.

**Table 1:** **Characteristics of liver biopsies specimens**.

ID	Diagnosis	Gender	Tissue collected (mg)	*IHL (×10^−3^)	^†^IHL yield (×10^−3^/mg)	^‡^IHL viability (%)	^§^NK cells (%)
1	1 y post LT for polycystosis	F	4	12	3.00	ND	ND
2	Steatosis of undetermined origin	F	22	140	6.36	ND	ND
3	Steatohepatitis, hepatic siderosis	F	11	60	5.45	ND	ND
4	5 y post LT for chronic hepatitis C	M	6	21	3.50	98.3	17.4
5	1 y post LT for sclerosing cholangitis	M	9	40	4.44	73.6	5.9
6	Possible NASH, steatosis	M	8	63	7.88	95.8	22.3
7	HCV cirrhosis	M	10	97	9.70	94.4	7.0
8	Polyarteritis nodosa, HCV	F	3	30	10.00	98.5	3.5
9	Chronic hepatitis B	F	9.6	120	12.50	93.1	16.7
10	HCV cirrhosis	M	11	130	11.82	93.0	4.5
11	Cirrhosis of undetermined origin	F	6.5	40	6.35	95.3	8.1
	Mean		9.1	68.5	7.36	92.7	10.7

*F, female; HCV, hepatitis C virus; LT, liver transplantation; M, male; NAFLD, Non-alcoholic fatty liver disease; NASH, non-alcoholic steatohepatitis; ND, not determinate*.

*The number of IHL was calculated based on the absolute number of isolated cells from liver tissue after dissociation and the percentage of lymphocytes obtained by flow cytometry FCS/SSC analysis.

*^†^The yield of IHL was calculated using the total number of IHL per mg of liver tissue*.

*^‡^IHL viability was determined by dead cell exclusion using PI staining by flow cytometry*.

*^§^NK cell percentages were obtained by gating on the CD3^−^CD56^+^ quadrant after exclusion of PI positive cells in the lymphocyte gate as defined by size and granularity in the FSC/SSC dot plot*.

### Isolation of intrahepatic lymphocytes

An enzymatic digestion method was utilized for the isolation of IHL modifying previously described protocols (Kawarabayashi et al., 2000; Morsy et al., 2005). In brief, hepatic tissue was washed thrice with HBSS containing 10% FCS to avoid blood contamination and to remove remaining intravascular lymphocytes. Samples were cut into 1 mm × 1 mm pieces with scissors in warm HBSS; then incubated for 30 min at 37°C with frequent shaking in buffer 1. Not-dissociated tissue and cell clumps were removed by filtration through a 30 μm nylon cell strainer (Miltenyi Biotec). To stop the enzymatic activity of the collagenase, the cells were washed twice at 400 × *g* during 10 min in 4°C cold HBSS containing 0.01 mg/ml DNase I. The cellular pellet was then resuspended in ACK lysing buffer for 2 min at room temperature to remove red blood cells, and then washed twice in AIM-V with 5% FCS. No additional lymphocyte isolation procedure was utilized to avoid any cell loss. Cells were used immediately for phenotypic flow cytometry assays and overnight stimulation in complete AIM-V media for functional analysis.

### NK cell phenotype analysis

**Figure 1:**
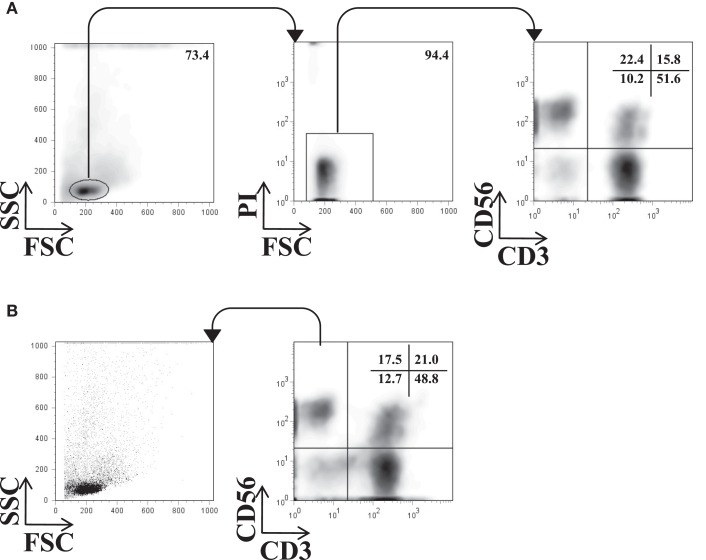
**Flow cytometry gating strategy for NK cells**. A representative example of the gating strategy used for the phenotypic analysis of IHL is shown. **(A)** Lymphocytes were identified by their size and granularity in the FSC/SSC dot plot (left), viability by excluding PI positive cells (middle), and NK cells by gating on the CD3^−^CD56^+^ quadrant. The phenotype of NK cells was then further analyzed by a panel of antibodies. **(B)** Back-gating control of all acquired CD3^−^CD56^+^ events in the FSC/SSC dot plot.

Peripheral blood mononuclear cells or IHL were resuspended in staining buffer, incubated for 30 min at 4°C with saturating concentrations of fluorescently labeled anti-CD3, -CD56, -CD16, -NKp46 mAb, and respective isotype-matched control antibodies, and analyzed by direct immunofluorescence on a FACS Calibur (BD Biosciences). The number of acquired events depended on the number of isolated cells and varied between 2,500 and 20,000. Lymphocyte gating was performed based on the forward and side scatter pattern; followed by dead cell exclusion using propidium iodide (PI) in all experiments. NK cells were localized in the CD3-negative and CD56-positive fraction (CD3^−^CD56^+^) (Figure 1A). To control whether substantial numbers of viable NK cells were excluded by the gating strategy described above, a back-gating control of all CD3^−^CD56^+^cells was used to trace them in the FCS/SSC dot plot (Figure 1B). Results were analyzed by using FlowJo software (Tree Star, Inc.) and presented as percentage of positive cells. To compare the levels of surface expression, the geometric mean fluorescence intensity ratios (MFIR) were calculated by dividing the mean fluorescence intensity (MFI) of staining with the mAb of interest with the MFI of the corresponding isotype control mAb.

### Flow cytometry-based NK cell functional assay

Peripheral blood mononuclear cells or IHL were resuspended at a concentration of 10^6^/ml in complete AIM-V medium. Different volumes of these suspensions were then seeded into 96-well V- or U-bottom plates (Nunc) and cultured overnight at 37°C in 5% CO_2_ and humidity in the presence of 50 U/ml of IL-2 and 0.5 ng/ml of IL-12. After careful removal of the culture media avoiding the removal the IHL, an equal volume of K562 cells resuspended in complete AIM-V medium was added at an NK:K562 ratio of 1:1 unless stated otherwise in the text. To obtain the number of NK cells present in the functional assays and to adjust the NK:K562 ratios to 1:1, viable PBMC or IHL were counted after isolation, a sample stained for CD3/CD56 and analyzed by FACS. The cell counts, the proportion of living cells in the lymphocyte gate, and the percentage of CD3^−^CD56^+^ NK cells were used to calculate the absolute numbers. The appropriate amount of K562 cells needed to obtain a NK to K562 ratio of 1:1 was then added to the co-cultures. Anti-CD107a mAb (1.25 μl/test) or isotype control antibodies (0.25 μl/test) were directly added to the culture wells at this time and the culture plate was spun at 100 × *g* for 1 min and placed in the incubator. Following stimulation with K562 cells for 1 h, GolgiStop (2 μM) was added and the cells were incubated for another 3 h at 37°C in 5% CO_2_. PBMC or IHL were then stained with anti-CD3 and anti-CD56 mAb for 30 min at 4°C. Samples were then fixed, permeabilized according to manufacturer’s instructions, and stained for intracellular IFN-γ for 30 min at 4°C. After washing, the expression of surface CD107a and intracellular IFN-γ was analyzed in the CD3^−^CD56^+^ NK cell fraction by four-color flow cytometry analysis.

### Statistical analysis

Values are expressed as mean or mean ± standard error of the mean (SEM) in the text and figures. The data were analyzed by a one-way ANOVA *F*-test, followed by Tukey–Kramer post test for multiple comparisons to evaluate each variable by pairs without pre-assignment of a reference condition. A probability value of *p* < 0.05 was considered to be statistically significant.

## Results

### Cell yields and viability of liver biopsy-derived intrahepatic lymphocyte

The different protocols published for the isolation of human IHL from LB are associated with variable cell yields and viability (Pham et al., 1994; Tran et al., 1997; Norris et al., 1998; Doherty et al., 1999; Kawarabayashi et al., 2000; Apolinario et al., 2002; Meier et al., 2005; Morsy et al., 2005; Tajiri et al., 2009). In the present work IHL were isolated by a combination of physical and enzymatic disruption of the liver tissue. Eleven LB specimens were analyzed their mean weight being 9.1 mg (range 3–22 mg). Following the liver dissociation, the viable isolated cells were counted by trypan blue exclusion and analyzed by flow cytometry. The percentage of IHL was obtained by gating on lymphocytes in the FCS/SSC dot plots and the total number of IHL calculated. As shown in Table 1, the mean viability of the IHL as measured by PI exclusion was 92.7% (range 73.6–98.5%), and the mean number of IHL isolated from 1 mg of liver tissue 7,364 (range 3,000–12,500 IHL/mg).

### Effect of collagenase IV and DNase I on NK cell viability and phenotype

As shown previously, enzymes such as collagenase IV and DNase I used for the isolation of IHL, may decrease the viability and expression of NK cell-surface markers such as CD56 (Curry et al., 2000; Morsy et al., 2005; Uhrberg, 2005). We therefore addressed the effect of these enzymes on the viability and surface expression of CD3, CD16, CD56, and NKp46 on PBMC and IHL, respectively. PBMC obtained from six healthy volunteers were incubated either in AIM-V media alone, buffer 2 containing 0.01 mg/ml DNase I, or in the presence of a combination of 0.5 mg/ml collagenase type IV (buffer 1) during 10, 20, or 30 min at 37°C. Moreover, hepatic tissue from five different liver resection specimens was homogenized and treated with a combination of collagenase type IV and DNase I for the same periods of time at 37°C. After RBC lysis and washing, enzyme-treated PBMC and IHL were immediately used for phenotypic analysis by flow cytometry.

**Figure 2:**
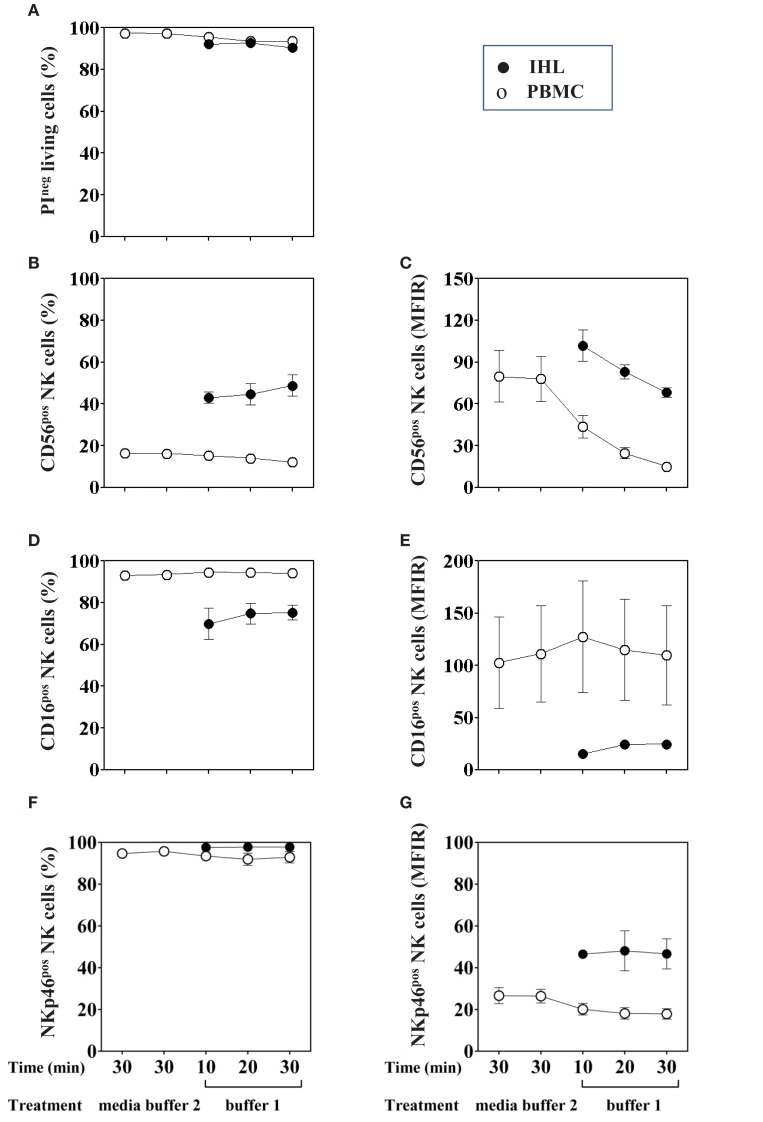
**Effect of collagenase IV and DNase I on NK cell viability and phenotype**. PBMC (open circles, *n* = 6) or homogenized liver tissue (filled circle, *n* = 5) were exposed to buffer 1 (containing collagenase IV and DNase I) for 10, 20, or 30 min at 37°C. As a control, cells were incubated in medium alone or buffer 2 (containing only DNase I). **(A)** Cell viability was evaluated by PI staining. **(B)** Percentage of CD56^+^ NK cells was determined by flow cytometry after gating on living lymphocytes. **(D)** Percentage of CD16^+^ and **(F)** NKp46^+^ cells was obtained by gating on CD3^−^CD56^+^ living lymphocytes. Mean fluorescence intensity ratio (MFIR) for **(C)** CD56, **(E)** CD16, and **(G)** NKp46 using the same gating strategy are also shown in the right column. Data are shown as mean ± SEM.

Viability was tested by PI exclusion staining and was shown to remain unchanged in both PBMC and IHL following incubation with the enzymes for up to 30 min compared to control cells that were exposed to media alone (Figure 2A). As to the expression of NK cell-surface markers, the percentage of peripheral blood CD56^+^cells was slightly decreased following 30 min of collagenase digestion (12.08 versus 16.45%), however, the difference did not reach statistical significance (*F* = 1.29, *p* = 0.3) (Figure 2B, open circles). Importantly, the percentage of intrahepatic CD56^+^ NK cells did not change when homogenized liver tissues were treated with collagenase and DNase for different time periods (Figure 2B, close circles). In addition, we confirmed that the percentage of CD3^+^ (data not shown), CD16^+^ and NKp46^+^ PBMC and IHL was unaffected after enzymatic treatment (Figures 2D,F, respectively). In contrast, MFIR of CD56 expression on both PBMC (Figure 2C, open circles, *F* = 5.16, *p* = 0.05) and IHL (Figure 2C, closed circles) was clearly reduced by enzymatic treatment, whereas CD3 (data not shown), CD16, and NKp46 expression levels did not change (Figures 2E,G).

Independently of the effect of the enzymatic treatment, the CD56, CD16, and NKp46 expression was shown to differ between PBMC and IHL. The percentages and MFIR of CD56^+^cells were higher in IHL than in PBMC, whereas the percentages and MFIR of CD16^+^ cells were higher in PBMC. Finally, the percentages of NKp46^+^ cells seemed to be equivalent in both, whereas the MFIR was higher in IHL. In summary, the analysis of phenotype and cell viability after enzymatic digestion treatment of PBMC and IHL did not show any differences in the percentages of NK cells, but it affected the level of CD56 surface expression.

### No effect of collagenase IV and DNase I on NK cell function

**Figure 3:**
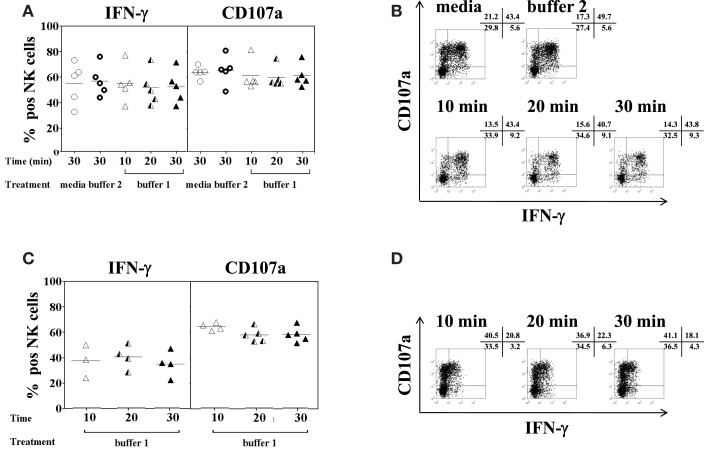
**Effect of collagenase IV and DNase I on NK cell function**. PBMC (*n* = 5) or homogenized liver tissue (*n* = 5) were exposed to 0.5 mg/ml collagenase IV (buffer 1) for 10 min (open triangles), 20 min (half triangles), 30 min (filled triangles), medium (open circles), or buffer 2 (rings) containing 0.01 mg/ml DNase I for 30 min at 37°C and incubated overnight in the presence of IL-2/IL-12 (50 U/ml and 0.5 ng/ml, respectively). Cells were then stimulated with K562 at a ratio of 1:1 for another 4 h. Intracellular IFN-γ production and CD107a surface expression on CD3^−^CD56^+^ NK cells were determined by antibody staining and flow cytometry. **(A)** Pooled data (*n* = 5) of the percentage of IFN-γ (left panel) and CD107a (right panel) expression on peripheral blood NK cells following enzyme exposure and activation and; **(B)** a representative example of IFN-γ and CD107a expression on peripheral blood NK cells. **(C)** Pooled data (*n* = 5) of the percentage of IFN-γ (left panel) and CD107a (right panel) expression on liver NK cells following enzyme exposure and activation and; **(D)** a representative example of IFN-γ and CD107a expression on liver NK cells. The mean values of five independent experiments are shown.

Limited information is available regarding the effect of enzymatic digestion used for the isolation of IHL on subsequent testing of NK cell functions, in particular degranulation and IFN-γ secretion. To this end, PBMC and liver resection samples were treated with 0.5 mg/ml of collagenase type IV and 0.02 mg/ml of DNase I for 10, 20, and 30 min, respectively; stimulated with IL-2/IL-12 overnight, co-incubated with K562 cells for additional 4 h, and analyzed by flow cytometry. Despite large inter-individuals variations, intracellular IFN-γ production and degranulation, as measured by CD107a surface expression (Alter et al., 2004; Uhrberg, 2005), of the NK cells present in PBMC did not differ between control and enzyme-treated samples, (Figures 3A,B; IFN-γ: *F* = 0.4, *p* = 0.8; and CD107a: *F* = 0.5, *p* = 0.8). Furthermore, no functional changes were observed in NK cells present in IHL isolated from liver resection specimens using different collagenase exposure times (Figures 3C,D). In general, intrahepatic NK cells seemed to produce less IFN-γ than peripheral blood NK cells although this difference did not reach statistical significance.

### Minimal cell numbers needed to detect the expression of CD16 on PBMC and IHL

**Figure 4:**
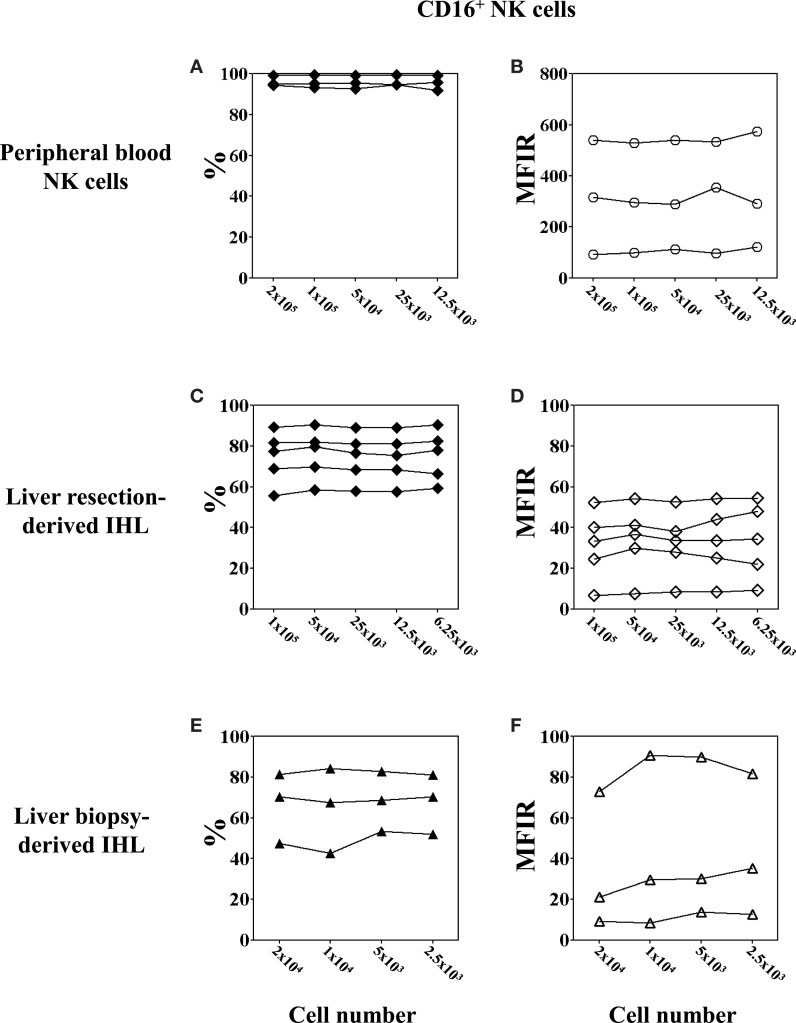
**Low cell numbers do not affect the flow cytometry results of CD16 expression on NK cell**. PBMC were obtained from healthy volunteers; IHL from liver resections and biopsies, respectively. Decreasing numbers of cells, ranging between 200,000 and 12,500 for PBMC, 100,000 and 6,250 for liver resection-derived IHL, and 20,000 and 2,500 for liver biopsy-derived IHL were used for the analysis. The expression of CD16 on peripheral blood and intrahepatic NK cells was determined by gating on CD3^−^CD56^+^ lymphocytes. **(A,B)** Peripheral blood NK cells, *n* = 3; **(C,D)** liver resection-derived IHL, *n* = 5; and **(E,F)** liver biopsy-derived IHL, *n* = 3. Data are expressed as percentage **(A,C,E)** and MFIR **(B,D,F)** of CD16^+^ NK cells.

Analysis of intrahepatic human NK cells is technically challenging given the very small number of IHL that can be obtained from needle LB. Therefore, the minimal number of cells necessary to assess the phenotype and function of peripheral blood and liver NK cells by flow cytometry was evaluated. As for the phenotype, CD16 was chosen because of its weak expression on IHL. First, we measured CD16 expression by multi-color flow cytometric analysis of the CD3^−^CD56^+^ NK cell population using different numbers of PBMC per tube, ranging from 200,000 down to 12,500 cells per staining and acquiring 20,000 when possible or 12,500 events. The results for the percentage of CD16^+^ NK cells (Figures 4A,C,E; closed symbols) was the same, irrespective of whether 12,500 PBMC (95.94%, range 91.77–99.31%), or 200,000 PBMC (96.21%, range 94.38–99.26%) were used in the labeling step (Figure 4A). Next, we evaluated CD16 expression on liver resection-derived IHL using different numbers, ranging from 100,000 down to 6,250 cells per tube and acquiring 20,000 events or less according to the number of cells in the tube. The results for the percentage of CD16^+^ NK cells using 100,000; 50,000; 25,000; 12,500; and 6,250 IHL per well, were 75.5, 79.7, 76.6, 75.4, and 78.1%, respectively. Thus, there were no significant differences using as few as 6,500 cell per tube for the analysis of CD16 expression as shown for five different donors (Figure 4C). At last, CD16 expression in LB-derived IHL obtained from three donors was analyzed. The percentages of CD16^+^ NK cells using 20,000; 10,000; 5,000–2,500 IHL per tube and acquiring all possible events was 66.44, 64.8, 68.32, and 67.79%, respectively (Figure 4E). In conclusion, the percentage of expression of CD16 on peripheral blood and intrahepatic NK cells can be reliably determined by flow cytometry using as few as 2,500 cells per tube containing as few as 352 (range 103–486) NK cells depending on the donor.

In order to further determine whether the total cell number used for the flow cytometry has an impact on the level of cell-surface marker expression, we assessed the MFIR of CD16 expression on peripheral blood and intrahepatic NK cells using different cell numbers for the analysis (Figures 4B,D,F; open symbols). The results for the MFIR of CD16 expression were identical regardless of whether 1,250 or 200,000 cells were used for the cell staining. This was true for both peripheral blood and intrahepatic NK cells, although large individual variations of CD16 MFIR levels were noted among the donors. Peripheral blood NK cells expressed CD16 with an MFIR ranging from 99.5 to 527.8 (mean 308) (Figure 4B), whereas the CD16 expression of intrahepatic NK cells was 10 times lower with an MFIR ranging from 6.7 to 52.2 (mean 31.4) for liver resection-derived IHL (Figure 4D), and from 11.0 to 83.8 (mean 41.3) for LB-derived IHL (Figure 4F).

Taken together, these results suggest that NK cell phenotype analysis by flow cytometry using very low cell numbers give comparable results to standard cell numbers. In addition, we confirmed that CD16 expression on liver NK cells is lower as compared to peripheral blood NK cells.

### Optimal NK to K562 ratio for NK stimulation in functional assays

**Figure 5:**
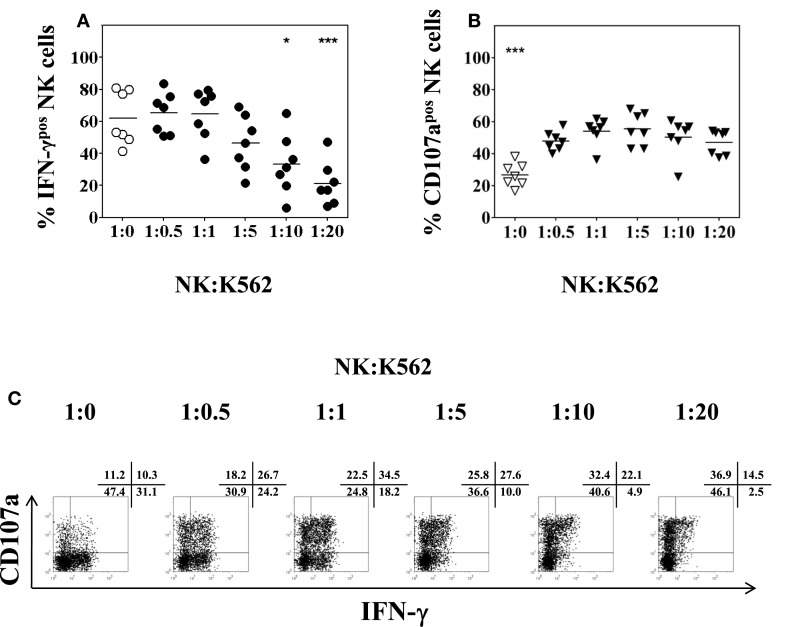
**Impact of the NK:K562 ratio on NK cell functional analysis**. PBMC isolated from healthy volunteers were incubated overnight in the presence of 50 U/ml IL-2 and 0.5 ng/ml IL-12, respectively. K562 cells were added for another 4 h at NK:K562 ratios of either 1:20, 1:10, 1:5, 1:1, or 1:0.5, taking into account the percentage of NK cells present in each PBMC isolation. **(A)** Percentage of IFN-γ positive CD3^−^CD56^+^ NK cells; and **(B)** percentage of CD107a expression of CD3^−^CD56^+^ NK cells as determined by flow cytometry following stimulation at different NK:K562 ratios. **(C)** A representative example of IFN-γ and CD107a expression of activated peripheral blood NK cells upon stimulation with IL-2/IL-12 overnight and different NK:K562 ratios is shown. Data of seven independent experiments are shown as mean and the *p*-values (*p < 0.05; ***p < 0.001) were calculated using one-way ANOVA test, followed by Tukey–Kramer post-test.

Whereas the production of intracellular IFN-γ by NK cells can be determined following overnight stimulation with IL-2/IL-12, the evaluation of NK cell degranulation by CD107a cell-surface expression, however, depends on additional stimulation with K562 cells using effector (PBMC) to target (K562) ratios of 10:1 or 5:1 (Alter et al., 2004; Meier et al., 2005; Aktas et al., 2009; Dessouki et al., 2010). In the present study, NK to K562 ratios rather than PBMC to K562 ratios were used to assess NK degranulation and IFN-γ production. To this end, the percentage of NK cells present in freshly isolated PBMC was measured by CD3 and CD56 staining. According to the resulting percentage, the desired NK:K562 ratio for the assays was adjusted. PBMC were incubated overnight in complete AIM-V medium containing 50 U/ml of IL-2 and 0.5 ng/ml of IL-12. K562 cells were added for another 4 h at NK:K562 ratios of 1:0.5, 1:1, 1:5, 1:10, and 1:20, respectively. As shown in Figure 5A, the percentage of IFN-γ positive CD3^−^CD56^+^ NK cells remained unchanged using NK:K562 ratios of 1:0.5 or 1:1 as compared to NK cells without K562 stimulation, but decreased significantly when NK:K562 ratios of 1:10 or 1:20 were used (*F* = 9.137, *p* < 0.001). This result indicated that it is important to determine the exact NK:K562 ratio in order to obtain reliable results for intracellular IFN-γ determinations. As to NK cell degranulation induced by K562 stimulation, the percentage of CD107a expression on CD3^−^CD56^+^ NK cells following co-incubation at different NK:K562 ratios, ranging from 1:0.5 up to 1:20, did not show any significant differences (*F* = 1.178, *p* = 0.3) (Figure 5B). A representative dot plot for the detection of intracellular IFN-γ and cell-surface CD107a expression is shown in Figure 5C. Taken together, anNK:K562 ratio of 1:1 was found to be optimal for stimulating NK cells prior to the detection of intracellular IFN-γ and cell-surface CD107a expression.

### Optimal culture volume and plate type for functional analysis of low numbers of peripheral blood and intrahepatic NK cells

**Figure 6:**
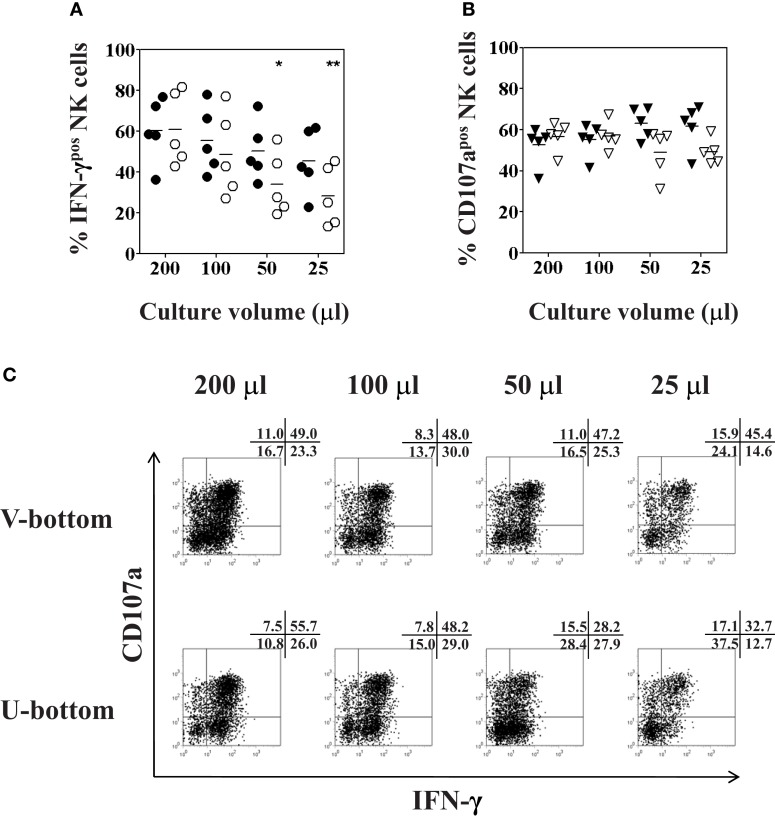
**Optimal culture volume and plate type for functional analysis of low numbers of peripheral blood NK cells**. PBMC isolated from healthy volunteers were resuspended in 200, 100, 50, or 25 μl of complete AIM-V medium at a constant concentration of 10^6^ cells/ml, and seeded in either 96-well U-bottom (open symbols) or V-bottom (filled symbols) plates overnight. K562 cells were added for another 4 h at a ratio of 1:1 for final stimulation. **(A)** Percentages of IFN-γ positive CD3^−^CD56^+^ NK cells (circles); and **(B)** percentages of CD107a expression (triangles) of CD3^−^CD56^+^ NK cells as determined by flow cytometry following culture in different culture volumes are shown. **(C)** Representative dot plot examples of IFN-γ and CD107a expression of NK cells cultured in different culture volumes as indicated, cultured either in U-bottom plates (lower panel) or V-bottom plates (upper panel). Data of five independent experiments are shown as mean and the *p*-values (*p < 0.05; **p < 0.01) were calculated using one-way ANOVA test, followed by Tukey–Kramer post-test.

To explore the best culture conditions requiring a minimal number of IHL to analyze NK phenotype and function, small culture volumes and different plate shapes were tested. First, PBMC were resuspended in complete AIM-V medium at a constant concentration of 10^6^cells/ml and seeded in 96-well U- or V-bottom plates at a total volume of 200, 100, 50, or 25 μl. Following overnight incubation flow cytometry analysis revealed a cell viability of near to 95% in all culture volumes. Moreover, there was no change of the percentage of CD3^−^CD56^+^ NK cells in all cultures, using as few as 25 μl of total volume (data not shown). Next, NK degranulation and intracellular IFN-γ production were determined following overnight culture using different volumes and different 96-well plate types and subsequent stimulation with K562 cells for another 4 h at a 1:1 ratio. The production of IFN-γ significantly decreased when U-bottom plates were used for the culture of small volumes (*F* = 3.53, *p* = 0.04), as shown in Figure 6A (open circles). More specifically, peripheral blood NK cells produced lower levels of intracellular IFN-γ when the PBMC were seeded in 50 or 25 μl of culture volume as compared to NK cells that were cultured in a volume of 200 μl (*p* = 0.03 and *p* = 0.01, respectively). On the other hand, incubation of PBMC in V-bottom plates in the same small culture volumes yielded higher values of IFN-γ production. Although there was also a trend toward a decrease of IFN-γ production using small volumes in V-bottom plates it was not statistically significant (*F* = 0.844, *p* = 0.5), as shown in Figure 6A (closed circles). As to the degranulation of NK cells, the culture volume did not affect the expression of CD107a on NK cells (Figure 6B), regardless of whether PBMC were seeded in small volumes, or whether they were seeded in 96-well U- or V-bottom plates (*F* = 1.5, *p* = 0.2 and *F* = 1.6, *p* = 0.2; respectively). Representative experimental dot plots are shown in Figure 6C.

**Figure 7:**
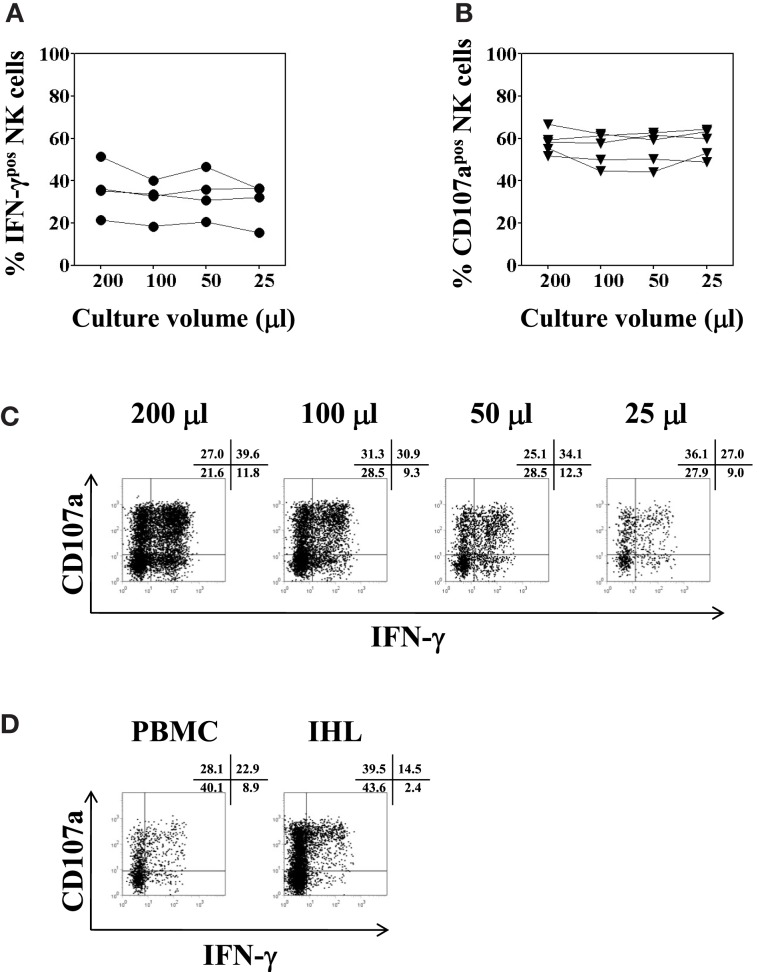
**Optimal culture volume for functional analysis of low numbers of intrahepatic lymphocytes**. IHL isolated from five different liver resection specimens were resuspended in 200, 100, 50, or 25 μl complete AIM-V medium, and seeded in 96-well V-bottom plates overnight. K562 cells were added for another 4 h at a ratio of 1:1 for final stimulation. **(A)** Percentages of IFN-γ positive CD3^−^CD56^+^ NK cells (filled circles, *n* = 4); and **(B)** percentages of CD107a expression (filled triangles, *n* = 4) of CD3^−^CD56^+^ NK cells as determined by flow cytometry following culture in different culture volumes are shown. **(C)** Representative dot plot examples of IFN-γ and CD107a expression of intrahepatic NK cells cultured in different culture volumes as indicated are shown. **(D)** Representative dot plot example of NK cell functional analysis using intrahepatic lymphocytes isolated from a liver biopsy is depicted, in parallel to the same assay using PBMC obtained from the same donor. IHL and PBMC were resuspended in 50 μl complete AIM-V medium, and seeded in 96-well V-bottom plates overnight. K562 cells were added for another 4 h at a ratio of 1:1 for final stimulation. Dot plots depict the respective percentages of CD107a and IFN-γ positive CD3^−^CD56^+^ NK cells.

To complete the analysis, IHL isolated from liver resection specimens were seeded in V-bottom plates in different culture volumes and tested for NK cell function as describe above. There were no significant differences between the various culture volumes used, neither for intracellular IFN-γ production (*F* = 2.6, *p* = 0.1), nor for CD107a expression (*F* = 1.2, *p* = 0.4), as shown in Figures 7A–C. Finally, a paired analysis of NK cell function in PBMC and IHL was performed acquiring a total number of each 25,000 PBMC and 38,000 LB-derived IHL total events obtained from the same donor. The cells were resuspended in 50 μl, seeded in a V-bottom plate, and incubated in complete AIM-V overnight before final stimulation with K562 cells for another 4 h at a 1:1 ratio. Whereas NK degranulation was found to be similar in peripheral blood and intrahepatic NK cells, the level of intracellular IFN-γ production was clearly higher in peripheral blood NK cells as compared to intrahepatic NK cells, 31.8 versus 16.9% in the representative experiment shown in Figure 7D.

Based on these results, we conclude that V-bottom plates are more suitable than U-bottom plates for overnight cultures in the presence of IL-2/IL-12 for the consequent determination of intracellular IFN-γ production in NK cells, in particular when small culture volumes and low cell numbers are used. By using V-bottom plates and keeping the concentration at 10^6^ cells/ml, it is possible to obtain NK functional assays with reliable results starting with a total volume of 25–50 μl, containing as few as 25,000–50,000 IHL.

## Discussion

The techniques for the isolation of single cell suspensions of IHL make use of mechanical and/or enzymatic dissociation of the tissue which usually is obtained from surgical resections (Curry et al., 2000; Morsy et al., 2005). Whereas mechanical methods have yielded very low cell numbers with low viability, a combination of gentle mechanical dissociation followed by digestion with collagenase type IV and DNase I resulted in higher yields of viable IHL suitable for phenotypic characterization by flow cytometry (Curry et al., 2000). In contrast to liver resection samples which depend on surgical interventions, standard biopsies consist of approximately 1/50,000 of the total mass of a liver, and are considered to be reasonably representative for diagnostic purposes (Bravo et al., 2001; Cholongitas et al., 2006). However, due to the small size of human needle LB specimen, the reported yields of IHL obtained from LB are very limited, ranging from 1.0 to 8.0 × 10^5^ cells (Tran et al., 1997; Apolinario et al., 2002; Meier et al., 2005; Tajiri et al., 2009). This wide range in cell yields is most likely the result of the different types and sizes of the needles used for the procedure, the number of biopsy passes, as well as the isolation techniques. In the present study, the IHL yield varied from 0.1 to 1.4 × 10^5^ cells (i.e., from 0.03 to 0.13 × 10^5^ IHL/mg of tissue), similar to the results of Morsy et al. (2005) who reported a yield between 0.08 and 0.10 × 10^5^ IHL/mg of tissue. Other groups had lower total yields (Tran et al., 1997; Apolinario et al., 2002; Meier et al., 2005; Tajiri et al., 2009), however, details on the weight of the LB were not given, and therefore it is not possible to compare the total cell number/mg of tissue.

### NK cell phenotype

Previous studies have found that DNase and collagenase preparations could affect the cell-surface expression of several markers, such as CD2, CD3, CD4, CD8, CD56, αβ, and γδ TCR (Mulder et al., 1994; Abuzakouk et al., 1996; Curry et al., 2000). However, the expression of these surface markers was largely unchanged when highly purified preparations containing less contaminating proteolytic enzymes, shorter exposure times, and lower concentrations were used (Norris et al., 1998; Morsy et al., 2005; Blom et al., 2009). In agreement with the latter findings, our results demonstrated that 30 min exposure to collagenase type IV (0.5 mg/ml) and DNase I (0.02 mg/ml) did not induce any difference in IHL viability, and the percentage of cells staining positively for CD56, NKp46, and CD16, respectively, stayed unaltered, as shown previously (Flynn et al., 1999). In contrast to the percentage and in line with an earlier report (Mulder et al., 1994), the level of CD56 surface expression, here reported as MFIR, was considerable diminished after the enzymatic treatment. Most importantly, we showed that the intracellular IFN-γ production and CD107a expression by NK cells was unaffected following 30 min of enzymatic treatment. Thus, human IHL isolated by a combination of mechanical dissociation and enzymes digestion were phenotypically and functionally representative.

Although flow cytometry cannot provide the information about the exact histopathological hepatic tissue distribution of IHL, it can determine the precise percentages of lymphocyte subsets which extend the information obtained by other means. Moreover, multi-color flow cytometry of IHL can identify their phenotype including activation markers in more detail than immunohistology (Doherty and O’Farrelly, 2000; Tajiri et al., 2009). Due to the very low IHL yields of LB specimens, a reliable method using minimal numbers of IHL was developed to assess phenotypic and functional characterization of intrahepatic NK cells. Previous investigators had evaluated the NK cell phenotype by gating on 1,000–5,000 IHL (Pham et al., 1994; Tran et al., 1997). Our flow cytometric analysis found that the CD16 expression on NK cells was unchanged by analyzing as few as 5,000 or even 2,500 total IHL (which correspond only to 644 and 352 counted NK cells, respectively) as compared to 100,000 IHL. In conclusion, it is possible to reliably characterize the phenotype of LB-derived NK cells by analyzing as few as 2,500–5,000 IHL per FACS tube. Using this minimal numbers we confirmed the findings of previous studies that intrahepatic NK cells express lower levels of CD16 and higher levels of CD56 as compared to peripheral blood NK cells.

### NK cell functional assay

To date only a few reports on intrahepatic NK cell degranulation and cytokine production have been published (Kawarabayashi et al., 2000; Zhang et al., 2011; Kramer et al., 2012; Varchetta et al., 2012). The small number of IHL available from LB and the numbers needed for functional analyses prevent purification protocols by cell sorting or magnetic isolation of intrahepatic NK cells as routinely performed for peripheral blood NK cells. In the present study we explored the minimal cell numbers and culture volumes required for reliable functional analysis of intrahepatic NK cells. The mean percentages of CD107a^+^ intrahepatic NK cells in response to stimulation with K562 target cells, and intracellular IFN-γ^+^ intrahepatic NK cells following overnight cytokine stimulation reached 60 and 40%, respectively. Our data also demonstrate that intracellular IFN-γ staining was significantly decreased when 50 or 25 μl cell suspensions at a constant concentration of 10^6^ cells/ml were seeded in 96-well U-bottom plates. In contrast, IFN-γ production remained unchanged when the same volume was incubated in V-bottom plates. These findings indicate that V-bottom plates are the optimal choice for small culture volumes to evaluate IFN-γ production by NK cells. This can be simply explained by the closer contact of cells in V-bottom plates as compared to U-bottom plates. Furthermore, stimulation at an NK to K562 ratio of 1:1 was found to be optimal for testing degranulation by CD107a surface expression. Intracellular IFN-γ production was clearly lower when K562 cells were added at higher ratios, indicating that K562 cells in excess influence the measurements negatively. We can only hypothesize on the underlying mechanism(s). The intracellular IFN-γ production of NK cells seems to attain maximal levels following overnight stimulation with IL-2/IL-12, whereas additional K562 stimulation for 4 h does not lead to a further increase of the ongoing production. In contrast, K562 stimulation which is necessary to induce degranulation may also induce IFN-γ secretion into the supernatant leading to the observed decrease of intracellular IFN-γ in a K562 cell number dependent manner. The NK to K562 ratio of 1:1 is optimal and in accordance with the original findings of Alter et al. (2004) demonstrating that intracellular IFN-γ and CD107a expression on NK cells can best be quantified simultaneously at PBMC to K562 ratios of 10:1, with NK cells accounting for about 10% of the PBMC. Our method differs only slightly from Zhang et al. (2011) who used a final NK to K562 ratio of 2:1 studying IHL that comprised nearly 20% of NK cells. They report between 10 and 40% of CD107a^+^ intrahepatic NK cells in response to K562 stimulation, higher values than found for peripheral blood NK cells, whereas intracellular IFN-γ production was low, on average 5%, following cytokine and K562 stimulation. In contrast, percentages of IFN-γ^+^ intrahepatic NK cells went up to 60% following PMA/ionomycin stimulation, with clearly lower values for peripheral blood NK cells (5–40%). Kawarabayashi et al. (2000) cultured IHL obtained from liver resections or PBMC on immobilized anti-CD3 antibodies, IL-2, or a combination of IL-2 and IL-12 and measured their IFN-γ production by ELISA, and the antitumor cytotoxicity by ^51^Cr-release assays. Compared to PBMC, they report a higher IFN-γ production and cytotoxicity against K562 target cells for IHL which decreased in samples obtained from cirrhotic livers. Using IHL obtained by mechanical disruption and further homogenization on a cell strainer from LB, the group of Nattermann (Eisenhardt et al., 2012; Kramer et al., 2012) recently reported intrahepatic NK cell degranulation of 10–25% in response to K562 stimulation, and inhibition of HCV replication *in vitro*. Finally, the group of Mondelli et al. (2010) reported a comprehensive analysis of the phenotype and function of intrahepatic NK cells obtained by mechanical disruption and enzymatic digestion (DNase 0.05 mg/ml; collagenase IV 0.5 mg/ml) in HCV infected patients as compared to healthy controls (Varchetta et al., 2012). Whereas the former study did not provide any details as to the yields of IHL isolation and the number of cells used for the functional assay, the latter gives information on the experimental protocol in the supporting materials. At least 7 × 10^5^ cells were plated in wells coated with the NKG2D ligand ULBP1 in the presence of IL-2 and IL-12 for the functional degranulation and cytokine secretion studies. The percentage of CD107a^+^ intrahepatic NK cells in response to K562 target cells was in average 35% for control and 25% for HCV infected patients, whereas our results showed 50–60% CD107a^+^ intrahepatic NK cells isolated from a heterogeneous population of patients. As to the production of intracellular IFN-γ, 5–10% were shown by Varchetta et al., whereas our stimulation method induced up to 50% IFN-γ^+^ intrahepatic NK cells. The percentage of intrahepatic NK cells able to secrete IFN-γ was lower than for peripheral NK cells under the same conditions while the degranulation of peripheral blood and intrahepatic NK cells after overnight cytokine stimulation was comparable. Taken together, using an optimized method to measure degranulation and intracellular IFN-γ production in low numbers of intrahepatic NK cells isolated from standard LB we confirm previous results on their CD107a expression but higher values for IFN-γ production either due to the culture conditions, which were optimized, or to the patient population studied.

In summary, characterization of intrahepatic NK cells in humans is technically difficult, given the very small number of lymphocytes that can be obtained from biopsies. In the present study, we have developed a reliable and efficient four-color flow cytometry method to evaluate the phenotype and function of intrahepatic NK cells by using IHL isolated from LB. In view of the recent advances in flow cytometry, this analysis can now be extended to up to 10 color analyses, thus increasing the amount of information collected from a LB. This novel method is especially suitable for analyzing needle biopsy samples, yielding only a few milligrams of liver tissue, without the need of major surgical interventions, since the phenotypic and functional analysis of LB-derived NK cells required only 5,000 per tube and 50,000 cells per culture well, respectively. This minimal amount is sufficient for reliable phenotypic and functional characterization of intrahepatic NK cells and will allow studies of IHL in larger cohorts which are critical for a comprehensive understanding of intrahepatic immune responses in liver diseases such as viral infections, autoimmune disease, and allograft rejection. Consequently, the establishment of this novel protocol should become highly valuable in attempts to elucidate the role of NK cells and other lymphocyte subsets in the pathogenesis of such liver diseases.

## Conflict of Interest Statement

The authors declare that the research was conducted in the absence of any commercial or financial relationships that could be construed as a potential conflict of interest.

## Abbreviations

IHL, intrahepatic lymphocytes; LB, liver biopsy; MFIR, mean fluorescence intensity ratio; NK, natural killer; PBMC, peripheral blood mononuclear cells.
